# Improving Breast Health Care in the State of Sergipe, Brazil: A Commentary

**DOI:** 10.1200/JGO.18.00114

**Published:** 2018-08-21

**Authors:** Catherine Duggan, Tauane A. Cruz, Maisa R.T. Porto, Cristiani L.M.S. Borges, Allison Dvaladze, Benjamin O. Anderson, Anna Cabanes

**Affiliations:** **Catherine Duggan**, **Allison Dvaladze**, and **Benjamin O. Anderson**, Fred Hutchinson Cancer Research Center, Seattle, WA; **Tauane A. Cruz** and **Anna Cabanes**, Susan G. Komen, Dallas, TX; **Maisa R.T. Porto**, MR Assessoria e Consultoria em Educacao e Saude, Lauro de Freitas, Bahia; and **Cristiani L.M.S. Borges**, Secretaria Municipal de Saude de Aracaju, Brazil.

Investments in the publicly funded health system—Sistema Unico de Saude (SUS)—and access to universal health care, guaranteed under the Brazilian Constitution of 1988,^1^ have resulted in significant improvements in the management of communicable diseases and decreases in maternal and infant mortality rates.^2^ Like other upper-middle-income countries, Brazil is experiencing an epidemiologic transition, where incidence and mortality rates from noncommunicable diseases, including breast cancer, have been steadily increasing. In 2004, the government of Brazil issued a Consensus Statement—Controle do Cancer de Mama: Documento de Consenso^3^—for the management of breast cancer. Prior studies have identified deficits in breast health care delivery, including dysfunctional referral pathways,^4^ low breast screening coverage,^5^ a high proportion of advanced-stage diagnoses,^6^ and deficits in treatment delivery.^7^ A study from 2012 examined changes in trends in breast cancer mortality in different states in Brazil. Although the authors reported a stabilization or decrease in breast cancer mortality rates in several states, an increase in mortality was observed in the Northeastern region: up 2.1% between 1980 and 2000 and up 5.3% from 2000 to 2009. In this region, Sergipe had the highest breast cancer mortality rate (12.5 per 100,000), corresponding to an annual percentage increase of 4.2%^8^ and an age-adjusted incidence rate of 58.89 per 100,000. ^9^

In response, the Breast Health Global Initiative (BHGI) at the Fred Hutchinson Cancer Research Center and Susan G. Komen (Dallas, TX), in collaboration with the Ministry of Health of Sergipe and the Municipal Secretary of Health of Aracaju, performed a standardized assessment of breast cancer health care delivery in the state of Sergipe, Brazil, located in the Northeastern coastal region of Brazil. The report, published on May 23, 2018,^10^ sought to review existing breast health care capacity, identify the relative strengths and weaknesses of the health system, and prioritize actionable items to advance breast cancer care in Sergipe

The baseline assessment and review of breast cancer early detection, diagnosis, and treatment was carried out from April to October of 2017. The research was based on methods developed by BHGI and assessed 18 primary, secondary, and tertiary care institutions in Aracajú, the State capital. Researchers interviewed breast cancer survivors to assess their experiences of the breast health care referral system and 35 primary health care providers.

The assessment identified a number of assets in Aracajú for breast cancer control: there is strong political support for the improvement of breast cancer, clinicians throughout the system are committed to delivering and improving breast cancer care in Sergipe, public-private partnerships provide pathology and imaging services to SUS patients to supplement services at public institutions, and tertiary care facilities provide appropriate locoregional and systemic therapies for treatment. However, many key challenges remain, which result in unclear and inefficient clinical pathways for women with breast health concerns and create significant delays in detection, diagnosis, and treatment. As a result, the relatively high proportion of late-stage disease reported at tertiary facilities is a cause for concern, with approximately 60% of women diagnosed at advanced stages (III or IV). Although treatment is free for all women, there is evidence that financial barriers and interruptions to drug supply hinder access to systematic treatment. Radiation therapy is only available in one of the tertiary level facilities surveyed in this assessment, and faulty equipment and low overall capacity cause significant delays in radiotherapy treatment.

Key recommendations of the report include:

- Effective breast cancer triage at lower levels of the health care system using clinical evaluation and clinical breast examination (CBE) of patients with breast symptoms and/or complaints can provide a significant opportunity to reduce delays in the diagnostic process if CBE is applied systematically, as recommended by the Consensus Statement; is performed by trained providers for women with breast health concerns; and is followed by organized referral to a secondary level facility within the SUS for diagnostic evaluation, including the use of breast ultrasound evaluation and, where appropriate, tissue sampling of suspicious breast lesions. Providers in primary level facilities should be educated on the signs and symptoms of breast cancer, trained in CBE, and supported in establishing and implementing referral protocols to higher levels of care.
- The patient referral system should be examined to assess what factors contribute to referral delays to highlight operational and system-specific barriers to rapid patient throughput; thus, opportunities for process improvement in organized patient navigation through the health system could be defined. Process improvement patient navigation projects should be piloted, examined, and, when successful, scaled up to reduce patient navigation delays.
- Strategic investment should be made in new equipment and technology, such as new radiotherapy units, costs that should be offset by increased and timely treatment completion rates, higher patient throughput, and less overall cost in ongoing repairs.
- An assessment of the underlying causes of chemotherapy and other medication shortages could help to identify key areas to target to address this problem, help streamline service delivery, and reduce burden on patients and on the health care facilities via increasing rates of successful completion of treatment, shortened wait times, and improvements in participant throughput.

Resource limitations exist across many health care settings and significantly affect breast cancer diagnosis and treatment, which in turn lead to poor outcomes for women. However organizational structure and barriers to care differ considerably across countries and regions: successful health systems are all alike (in that they reduce mortality from breast cancer), but every dysfunctional health system is dysfunctional in its own way. Thus, guidelines such as the BHGI’s and the National Comprehensive Cancer Network’s resource-stratified guidelines, which provide a framework for organizing breast health care in the context of available resources,^11^ must be considered not only in the context of available resources but also in terms of the current organization of the health system and barriers and facilitators that are specific to that system. To implement meaningful changes, an organized approach is needed, beginning with a situation analysis to identify key gaps that prevent patients from flowing through the system and receiving necessary services. Identification of system strengths and deficits should form the basis of comprehensive plans to address specific needs, so that systems can be built in ways that are functional and sustainable, as a basis for improving patient outcomes.
